# Epidemiology, risk factors, diagnosis, and treatment of intra-abdominal traumatic neuromas - a narrative review

**DOI:** 10.1186/s12876-023-03049-y

**Published:** 2023-11-28

**Authors:** Yaoqun Wang, Sishu Yang, Bei Li, Cunyong Shuai, Xianze Xiong, Jiong Lu

**Affiliations:** 1 Division of Biliary Tract Surgery, Department of General Surgery, West China Hospital, Sichuan University, Chengdu, Sichuan 610041 China; 2 Research Center for Biliary Diseases, West China Hospital, Sichuan University, Chengdu, Sichuan 610041 China; 3 Department of Hepatobiliary Surgery, Sichuan Provincial Corps Hospital, Chinese People’s Armed Police Forces, Leshan, China

**Keywords:** Traumatic neuroma, Epidemiology, Risk factors, Diagnosis and treatments

## Abstract

Traumatic neuroma (TN) is a disorganized proliferation of injured nerves arising from the axons and Schwann cells. Although TN rarely occurs in the abdominal cavity, the incidence of TN may be underestimated because of the large number of asymptomatic patients. TN can cause persistent pain, which seriously affects quality of life. TN of the biliary system can cause bile duct obstruction, leading to acute cholangitis. It is difficult to differentiate TN from malignancies or recurrence of malignancy, which results in a number of patients receiving aggressive treatment. We collected cases reports of intra-abdominal TN over the past 30 years form PubMed and cases diagnosed in our medical center over the past 20 years, which is the largest case series of intra-abdominal TN to the best of our knowledge. In this review, we discuss the epidemiology, pathophysiology, risk factors, classification, diagnosis, and management of intra-abdominal TN.

## Highlights

This is the largest case series and case review of traumatic neuroma in the abdominal cavity.

We conclude and update the clinical and epidemiological characteristics of TN in the abdominal cavity.

We assessed and discussed the management of TN in the abdominal cavity, especially TBN.

## Introduction

Traumatic neuroma (TN) is not a true neoplasm but an abnormal proliferation of injured nerves with scar tissues resulting from trauma, surgery, bleeding, or ischemia [1]. It may occur anywhere but is most common in the lower extremities, followed by the head and neck [2]. The first TN was reported by Odier in 1811 with veterans suffering from disabling pain in their amputated limbs [3, 4]. TN in the digestive system is rare and mostly occurs in the biliary tree after cholecystectomy or liver transplantation, known as traumatic biliary neuroma (TBN), because of the abundant nerve supply to the gallbladder, cystic duct, and hepatic ducts [5]. The first case involving the digestive system was described in 1928 by Husseinoff [6]. Most intra-abdominal TNs are asymptomatic [7, 8]. Some TBNs in cystic duct after cholecystectomy may cause biliary-type pain and result in post cholecystectomy syndrome [9, 10]. Only a few patients develop acute cholangitis or fatal graft dysfunction due to obstruction by a neuroma, presenting with jaundice [11–15]. Other sites reported including celiac trunk, ampulla of Vater, pancreas, inferior mesenteric artery stump and rectal wall, etc. [2, 7, 8, 16, 17]. Although TN is a benign lesion, it is sometimes difficult to differentiate it from a malignant tumor, and some patients receive aggressive treatment. In this review, we collected a total of 93 cases of intra-abdominal TNs reported in the past 30 years in the literature and those diagnosed in our medical center in the past 20 years and determined the clinical and pathological characteristics and diagnostic and therapeutic approaches for intra-abdominal traumatic neuroma.

## Epidemiology

Owing to the lack of effective methods for preoperative diagnosis and the large proportion of asymptomatic patients, the incidence of TN may be underestimated [9]. Only a few European studies have reported the incidence of TBN after cholecystectomy or liver transplantation; the rest of the TNs in the abdomen are sporadic cases [2, 8, 17, 18].

One study reported that TBN was found in approximately 10% post cholecystectomy patients during autopsy [19]. Another postmortem study suggested that remarkable nerve proliferation in the remnant cystic duct was observed in almost 40% post cholecystectomy patients, 28% of whom had TBN [20]. In this review, we collected 32 cases of TBN after cholecystectomy (Table 1), including 4 patients who underwent laparoscopic cholecystectomy and 4 patients who underwent common bile duct exploration. The ratio of men to women was nearly 3:1 (23 males, 8 females and 1 unknown). The incidence is higher in males than females, which is in contrast to the fact that women develop cholecystolithiasis more often than men [21]. The incidence of TBN increases with age, nearly 70% patients are > 60 years of age. The median age at diagnosis was 64 years (range:39–81 years). The interval between cholecystectomy and the diagnosis varied from 2 months to 56 years. The median interval between the open cholecystectomy and TBN was 17 years. Over 65% patients diagnosed above 10 years after open cholecystectomy, which is consistent with another study’s result that the median time from surgery to diagnosis is more than 12 years in the subgroup of patients who underwent open cholecystectomy [12]. Compared with open cholecystectomy, rare cases have been reported after laparoscopic cholecystectomy. On the one hand, patients who receive open cholecystectomy usually suffer from severe cholecystitis or other complications, which may increase the difficulty of the surgery and risk of damaging bile ducts, nerves and arteries. However, given the long time from TBN formation to symptom onset, it is premature to conclude that the incidence of TBN after laparoscopic cholecystectomy is lower than that after open cholecystectomy.

**Table 1:** Published case reports of TBN over the past 30 years and cases in our medical center over the past 20 years

Author	No.	Sex	Age (years)	First surgery	Clinical presentation	Post-surgery	Interventional procedure	TN treatment	Neuroma size,mm
Yasuda et al. [67]	1	Male	76	OC	-	56 years	-	Observation	14
Lalchandani et al. [12]	2	Male	41	OC + BDI	Epigastric pain	10 years	ERCP + stent	Res + HJ	-
Toyonaga et al. [58]	3	Female	76	OC	-	46 years	-	Observation	8
Kim et al. [10]	4	Male	76	OC	Alteration of CA19-9	17 years	-	Res + HJ	10
Paquette et al. [28]	5	Female	71	OC + CBDE	Jaundice	45 years	-	Res + HJ	20
Choi et al. [1]	6	Male	46	OC	LFT alteration	-	-	Right hemi-hepatectomy	20
Ueno et al. [5]	7	Male	60	OC	Jaundice	18 years	-	Res + HJ	-
Capovilla et al. [45]	8	Male	60	OC + BDI	Jaundice	3 months	-	Res + HJ	-
Topazian et al. [73]	9	Female	45	LC	Abdominal pain	1 month	EUS-guided nerve block	Resection	22
Hotta et al. [11]	10	Male	60	OC + CBDE	Jaundice	17 years	-	Res + HJ	-
Iannelli et al. [13]	11	Male	81	OC	Jaundice	12 years	-	Res + HJ	-
Shimura et al. [57]	12	Female	70	OC	Abdominal discomfort	22 years	-	Res + HJ	11
Shumate et al. [32]	13	Male	68	OC	Epigastric pain	29 years	-	Right hemi-hepatectomy + Res + HJ	30
Chantranuwat et al. [47]	14	Male	70	OC	Jaundice	-	-	Resection	20
Hyman et al. [52]	15	-	-	OC	Jaundice	3 years	ERCP + stent	Res + HJ	-
Nagata et al. [48]	16	Female	53	LC	Jaundice	2 months	-	Res + HJ	-
Nagafuchi et al. [49]	17	Female	39	LC	Jaundice	8 months	-	Res + HJ	-
Saint-Paul et al. [50]	18	Male	64	OC	Jaundice	4 years	-	Res + HJ	-
Pickens et al. [19]	19	Female	65	OC	Abdominal pain	40 years	-	Res + HJ	-
Koh et al. [76]	20	Male	70	OC HJ	-	25 years	-	PPPD	-
						12 years	-		
Kim et al. [68]	21	Male	72	OC	-	30 years	-	Resection	18
**Our center**	22	Male	62	OC + CBDE	Jaundice	13 years	-	Whipple operation	23
	23	Male	55	OC	Abdominal pain	6 years	-	Whipple operation	-
	24	Male	53	OC + CBDE	LFT alteration	3 years	-	Res + HJ	-
	25	Male	75	LC	Jaundice	4 months	-	Res + HJ	-
	26	Male	58	OC	Abdominal pain	8 years	-	Res + HJ	20
	27	Male	62	OC	Jaundice	12 years	-	Res + DD	15
	28	Male	51	OC	Jaundice	20 years	-	Whipple operation	30
	29	Male	55	OC	Jaundice	21 years	-	Resection	11
	30	Female	33	CCC resection + HJ	Jaundice	6 years	-	Res + HJ	-
Yang et al. [27]	31	Male	65	Left hemi-hepatectomy	Jaundice	8 years	-	Res + HJ	15
Cheng et al. [77]	32	Male	68	HICC resection + HJ	Jaundice	3 years	-	Res + HJ	17

OC ~ open cholecystectomy; LC ~ laparoscopic cholecystectomy; Res + HJ ~ resect stricture of bile duct + hepaticojejunostomy; Res + DD ~ resect stricture of bile duct + duct to duct biliary anastomosis; LFT ~ liver function tests; BDI ~ bile duct injury; CBDE ~ common bile duct exploration; CCC ~ congenital choledochal cyst; HICC ~ hilar cholangiocarcinoma; PPPD ~ pylorus-preserving pancreaticoduodenectomy

Nine patients from our center were included in this study (8 males and 1 female). Patients who undergo cholecystectomy in our hospital receive regular follow-up, and the maximum follow-up time for asymptomatic patients is 10 years. Among these nine patients, five had TN during follow-up. Four patients showed no changes during the follow-up period, but were found to have TN after more than 10 years of follow-up and were treated again in our hospital. The patients ranged in age from 51 to 75 years, with a minimum onset time of 4 months and a maximum of 21 years after surgery. Seven patients had previously undergone open cholecystectomy, one had previously undergone laparoscopic cholecystectomy, and one had previously undergone resection of a congenital choledochal cyst. Abdominal pain was the main clinical manifestation in six patients, 2 patients, abdominal pain in two patients, and abnormal liver function in one patient.(Table 1).

Biliary stenosis is one of a common complication of liver transplantation (LT), with an incidence of 5% ~ 28% after deceased-donor transplant and 28% ~ 37% after living-donor transplant [22, 23]. A study from France reported that symptomatic and histologically proven TBN accounted for 9.6% of anastomotic biliary stenosis [24], which is similar to the study from Croatia on TBN, representing 6.1% of liver re-transplantation [15]. As for the incidence of TBN after LT, the results vary from 0.6 to 9.2% in different studies (Table 2) [9, 15, 25, 26]. When it comes to the symptomatic TBN, the incidence is even lower from 0.5% ~ 2.8% [9, 25]. There are totally 56 cases in this review (Table 2). Similar to TBN after cholecystectomy, the incidence was much higher in male than female with a ratio at 4 of 1. The interval between the diagnosis of TBN and first transplantation ranged from 1 to 239 months. Although the time span is quite long, more than 50% TBN diagnosed within one year of the first transplantation. The median time to diagnosis of TBN varies from 6 to 69 months [15, 24, 25]. There is discrepancy in the median time from surgery to diagnosis after liver transplantation of different studies, but it’s notably shorter than neuromas diagnosed after cholecystectomy [9, 15]. Among the 30 patients with certain types of biliary reconstruction, only one underwent hepaticojejunostomy and the rest underwent duct-to-duct anastomosis.

**Table 2:** Published case reports of TN following LT over the past 30 years

Author	Incidence	No	Sex	Age (years)	Type of biliary reconstruction	Clinical presentation	Post-Tx, (months)	Interventional procedure	TN surgical treatment	Neuroma size,mm
Mrzljak et al. [15]	6.1%*	1	Male	54	D-D	Jaundice	43	None	Re-LT	15
		2	Female	32	D-D	Jaundice	10	None	Re-LT	17
		3	Male	54	H-J	Abdominal discomfort	51	None	Re-LT	30
		4	Male	58	D-D	Recurrent dilatation	3	ERCP, balloon dilatation	Re-LT	30
		5	Male	60	D-D	Jaundice	49	None	Re-LT	25
		6	Male	64	D-D	Jaundice	17	Biliary drainage	Re-LT	30
		7	Male	60	D-D	Jaundice	16	None	Re-LT	20
Terzi et al. [78]	-	8	F	17	D-D	Cholangitis	3	Plastic stenting	Res + HJ	16
Navez et al. [24]	0.5%	9	-	-	D-D	Bile leakage	239	None	Res + DD	6–35^#^
		10	-	-	D-D	Cholangitis	162	None	Res + HJ	-
		11	-	-	D-D	Cholangitis	69	ED	Res + HJ	-
		12	-	-	D-D	Jaundice	31	None	Res + HJ	-
		13	-	-	D-D	LFT alteration	4	PTHD	Res + HJ	-
Herrera et al. [25]	3.5%	14	12 Males 3Females	16–65^#^	D-D	Jaundice	6	None	Res + DD	-
		15	-	-	D-D	Jaundice	9	None	Re-LT	-
		16	-	-	D-D	Jaundice	17	None	Excision	-
		17	-	-	D-D	Jaundice	2	Balloon dilatation	Res + DD	-
		18	-	-	D-D	Jaundice	12	None	Res + DD	-
		19	-	-	D-D	Jaundice	9	Balloon dilatation	Res + DD	-
		20	-	-	D-D	Jaundice	1	None	Res + DD	-
		21	-	-	D-D	Jaundice	12	None	Re-LT	-
		22	-	-	D-D	Jaundice	2	None	Res + DD	-
		23	-	-	D-D	Jaundice	18	Balloon dilatation	Res + HJ	-
		24	-	-	D-D	LFT alteration	4	none	Res + HJ	-
		25	-	-	D-D	Jaundice	2	Balloon dilatation + stent	Res + HJ	-
		26	-	-	D-D	LFT alteration	1	Balloon dilatation	Res + DD	-
		27	-	-	D-D	Jaundice	1	None	Re-LT	-
		28	-	-	D-D	LFT alteration	4	None	Res + DD	-
Mentha et al. [26]		29	M	59	D-D	LFT alteration	17	Balloon dilatation	Res + HJ	10
		30	M	46	D-D	Bile duct stenosis	5	Balloon dilatation + stent	Res + HJ	-
Colina et al. [9]	27.9%	31–56	-	-	-	-	3–25^#^	-	-	10–25^#^

D-D ~ duct to duct biliary anastomosis; H-J ~ hepaticojejunostomy; Res + HJ ~ resect stricture of bile duct + hepaticojejunostomy; Res + DD ~ resect stricture of bile duct + duct to duct biliary anastomosis; LFT alteration ~ liver function tests; ED ~ endoscopic drainage; PTHD ~ percutaneous transhepatic drainage

*for liver re-LT in authors’ institution; # range from patients reported

TN also occurres after other abdominal surgeries, including gastrectomy, polypectomy and, rectal cancer surgery(Table 3) [2, 7, 8, 27]. One patient did not have a history of surgery, but suffered from blunt abdominal trauma [18].

**Table 3:** Published case reports of TN with other sites intra-abdominal

Author	No.	Sex	Age (years)	First surgery	Location	Post-surgery	TN treatment	Neuroma size,mm
Jeon et al. [2]	1	Male	59	U-LAR Colo-anal anas	Stump of inferior mesenteric artery	32months	Resection	18
Kwon et al. [7]	2	Male	56	Distal gastrectomy Subtotal gastrectomy	Celiac trunk	9years 5months	Resection	35
Curran et al. [8]	3	Male	53	Endoscopic polypectomy	Rectum	6years	Resection	39
Furukawa et al. [51]	4	Male	76	Distal gastrectomy	Remnant stomach	13years	ESD	18
Estifan et al. [69]	5	Female	50	-	Rectum	-	Resection	4

U-LAR ~ ultra-low anterior resection

## Pathophysiology mechanism & risk factors

Normally, the continuity between the two ends of a severed nerve is re-established by the orderly growth of axons from the proximal to the distal stump through tubes of proliferative Schwann cells. When the nerve ends are far apart or missing stumps, which prevent the reestablishment of neural continuity, hyperplastic proliferation of axons mixing with Schwann cells in a fibrocollagenous stroma develops into TN at the proximal end of the injured nerve [26]. The mechanism of this dysregulating growth pattern still remains unclear, athough a few studies have reported increasing levels of fibroblast growth factor and its receptor in TN [19, 28]. Targeting the pathophysiology of TN, He et al. found that chondroitin sulfate proteoglycans (CSPGs) can inhibit the formation of TN by blocking irregular axon regeneration in the proximal nerve stump. Kryger et al. found that trkA-IgG (an inhibitor of nerve growth factor) can reduce the information of TN in a rat model, but further research is still needed [29, 30].

Surgery is the primary risk factor for TN. Surgical manipulations, including excessive exploration, thermocoagulation, and vascular ligation, which may damage the surrounding nerves or arteries, can increase the risk of TN [1]. As many sympathetic and parasympathetic nerves are located outside the wall of the bile ducts, most TBN are extraluminal. However, if the common duct or hepatic duct is damaged during a careless or difficult surgery, intraluminal proliferation of nerves associated with an inflammatory scar can occur [31, 32], which may cause bile duct obstruction at the early stage after surgery. Three patients (two with laparoscopic cholecystectomy and one with open cholecystectomy) had bile duct injury during cholecystectomy and were diagnosed with TBN within 3 months, which was significantly different from the long interval time of cholecystectomy. With progress in laparoscopic techniques, laparoscopic cholecystectomy has been applied to a wider range of patients. When performing difficult laparoscopic cholecystectomy for acute cholecystitis, bailout procedures could be helpful in preventing bile duct injury, which theoretically decreases the incidence of TBN [33].

The situation is much more complicated with regard to TBN after LT. The origin of TBN after transplantation is still controversial, with some suggesting that it arises from the recipient bile ducts because most TBNs reported after LT occurred in patients who had duct-to-duct biliary reconstructions and less frequently occurred after bilioenteric reconstruction, since nerve regeneration originated from the proximal nerve ending [5, 24]. Others considered that it may arise from the recipient, or from the donors’ nervous tissue, on account of the small bifurcating nerve trunks seen in the perihilar intrahepatic septa during histological examinations of allografts with hilar neuromas, which indicates the survival of donors’ innervation [9, 26].

A few studies found that the number of nerve fibers decreased immediately after severing of the main hilar trunks; then, it increased due to proliferation and reinnervation in the post-transplant period [34, 35]. This result is consistent with the fact that the incidence of TBN is much higher in patients more than 3 months after transplantation [9]. Immunosuppressors may play an important role in accelerating nerve proliferation and reinnervation. Tacrolimus was found that could improve neurologic recovery and enhance axon regeneration by its neurotrophic and immunosuppressive actions after peripheral nerve injures [36], what’s more, the another common immunosuppressor, cyclosporine, was also found that had a promotion in axon growth of the recipient proximal nerve endings into nerve allografts in rats [37]. Therefore, immunosuppressants may be risk factors for TBN.

Besides surgical manipulations and immunosuppressants, infections, foreign bodies, trauma, ischemia, and scarring may also contribute to the formation of TBN [18, 38]. The continuity of the nerve can also be inhibited by granulation and fibrous tissue arising from the surrounding blood vessels and adjacent soft tissues [26]. There are also a few neuromas in the bile duct without any history of surgery or trauma; it might be postulated that bile or cholesterol are the inciting stimuli for fibrous and neural proliferation [31, 38].

## Classification

There are several classification methods that are based on various factors. Nerve continuity is the most commonly used method. End-bulb neuroma (EBN), also known as terminal neuroma or stump neuroma, is a bulbous enlargement from the end of a completely disrupted nerve. Neuroma-in-continuity (NIC), also called spindle neuroma, results from partial nerve transection [39–43], and is divided into two pathological types: spindle neuromas with intact perineurium or lateral neuromas that occur after partial disruption of the perineurium and after nerve repairs [44]. This is similar to the results of Colina et al. that if the perineurial continuity of injured axons is preserved, encapsulated neuroma occurs macroscopically as small white-gray nodules macroscopically [9, 10] If the continuity of the perineurium is interrupted, branching axons would invade the mesenchyme, resulting in uneven thickening of duct walls [9]. Herrera et al. classified TBN into two types according to its pathological characteristics and location: type I originates from and is located in the main biliary tract wall, while type II originates from the surrounding tissues next to the main biliary tract [25]. They suggested that this type of classification is useful for treatment decision making.

## Diagnosis

### Clinical manifestation

When the intraluminal TBNs occur or extraluminal TBNs cause obstruction in common bile duct, the most common presentation is jaundice [5, 27, 45–50]. Several of patients are more likely to present with right upper quadrant pain, elevated transaminase levels and anorexia [2, 19]. Several studies have suggested that TBNs may be blamed for post cholecystectomy syndrome because of the relief of symptoms after surgical resection in most cases [10]. Given that many nerves normally surround the extrahepatic bile duct, the number of symptomatic patients was lower than that expected. Some patients are asymptomatic, and lesions can be detected accidentally on radiological examinations [7, 51].

### Accessory examination

#### Laboratory examination

Patients with TBN may have abnormal laboratory results due to biliary obstructions, such as elevated bilirubin and transaminase levels. Patients sometimes present with elevated levels of carbohydrate antigen 19 − 9 (CA19-9) due to cholangitis [28]. Nevertheless, the magnitude of CA19-9 elevation cannot be used as a specific indicator to differentiate biliary malignancies from TBN [52]. A previous study suggested that patients with benign tumors had a lower elevated level of CA19-9, which returned to normal after the relief of obstruction compared to malignant diseases [53], which may aid in the differential diagnosis.

#### Imaging examination

Although it is difficult to diagnose TN preoperatively, imaging remains an indispensable component of the diagnosis. This method requires further treatment. Few studies have described the imaging characteristics of TN in the abdominal cavity; hence, in addition to summarizing cases in our center, we learned from TN located in the limbs, head, and neck. The imaging characteristics of the TN are shown in Table 4.

**Table 4:** Imaging characteristics for TN.

Imaging examination	Traumatic neuroma (TN)	
	TN located in abdominal cavity	TN located in limbs, head and neck
Ultrasonography	-	1. Smaller short axis diameters and short-to-long axis ratios than recurrent LN; 2. Absence of vascular flow; 3. Most of TNs were fusiform; 4. Well-defined or ill-defined margin; 5. Central hyperechogenicity; 6. Internal linear hypoechogenicity .
CT	Enhanced mass with central hypoattenuation and hyperattenuating rim	Nodules with central hypoattenuation and hyperattenuating rim
	The appearance of TN on CT could be various and may be location-related	
MRI	T1:Heterogeneous thicken of the common bile ducts with contrast enhancement; T2: Markedly homogeneous or heterogeneous enhanced nodules with low-intensity capsule.	T1: Homogeneous nodules iso-intense to muscles; T2: High-intensity with hypointense rim and heterogeneous contrast enhancement.
PET/CT	The diagnosis of TN can’t be excluded just by a positive results of PET/CT.	
EUS	A homogeneous hypoechoic mass with a clear margin.	-
IDUS	TN located at cystic stump.	-
ERCP	TN were covered by normal bile duct mucosa.	-

EUS ~ endoscopic ultrasonography; CT ~ Computed tomography; MRI ~ Magnetic resonance imaging; PET/CT ~ Positron emission tomography/computed tomography; IDUS ~ Intraductal ultrasonography; ERCP ~ endoscopic retrograde cholangiopancreatoscopy

##### Ultrasonography

Several studies have analyzed the characteristics of TN on ultrasonography to aid in the differential diagnosis between TN and recurrent lymphadenopathy after neck dissection. All concluded that TN had smaller short-axis diameters and shorter to long-axis ratios than recurrent lymphadenopathy [54, 55]. Moreover, the absence of vascular flow is another important characteristic of TN compared with recurrent lymphadenopathy. Most of TNs were fusiform, the rest were oval [55, 56]. As for the margin, one study suggested that TN had well-defined margins [56], while another study considered that TN had ill-defined margins [55]. Different types of TN may account for these differing results. When a capsule occurs, it may have a well-defined margin; otherwise, it may have an ill-defined one. The presence of central hyperechogenicity, which results from dense collagenous tissue, is also considered one of the sonographic features of TN [54, 56]. The nerve from which a TN originates may exhibit internal linear hypoechogenicity [55].

##### Computed tomography (CT)

**Fig. 1:**
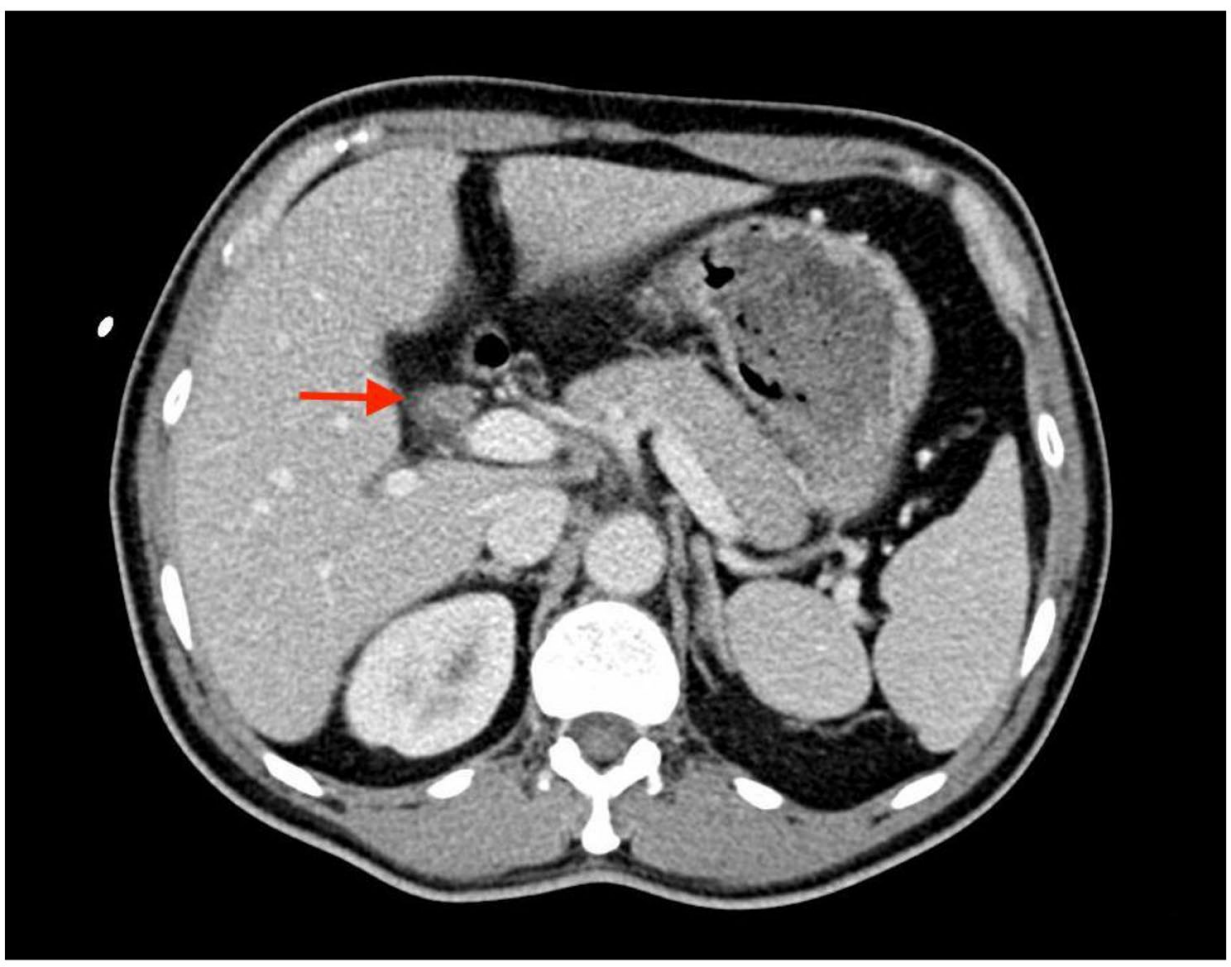
Portal-venous phase CT shows dilation of the bile ducts and an enhanced mass with central hypoattenuation and hyperattenuating rim

TBNs can be contrast-enhanced on CT imaging [5, 10, 57, 58], which is consistent with the results of our study (Fig. 1). TNs after neck dissection could also appear as nodules with central hypoattenuation and a hyperattenuating rim [54, 59]. This suggests that the appearance of TN on CT can vary and may be location-related. Neither contrast enhancement nor a hyperattenuating rim can be used as a differentiating characteristic because they are also observed in malignancy and recurrent lymphadenopathy. A previous case of TN around the stump of the inferior mesenteric artery described the dynamic process from an irregular-margin lesion to a well-circumscribed nodule with enlargement of the diameter [2], which is not typical for TN and usually remains stable over the years [59].

##### Magnetic resonance imaging (MRI)

TNs in the limbs or neck appear as homogeneous nodules isointense to muscles on T1-weighted images, high-intensity with hypointense rims on T2-weighted images, and heterogeneous contrast enhancement [42, 54, 60]. Few studies have described the characteristics of intra-abdominal TN on MRI. It is difficult to detect TNs located in the biliary tree because of the same signal intensity between the nerves, soft tissues, and pancreatic head [31]. Some studies found only bile duct dilatation on MRI without a compressive mass [11, 31]. Several studies detected markedly homogeneous or heterogeneously enhanced nodules with low-intensity capsules on T2-weighted images [2, 5, 24]. Some authors have suggested that the surrounding fibrous scar tissues of TN correspond to the hypointense rim, mimicking a capsule, which was observed by histopathologic examination of the specimen [54, 61].

**Fig. 2:**
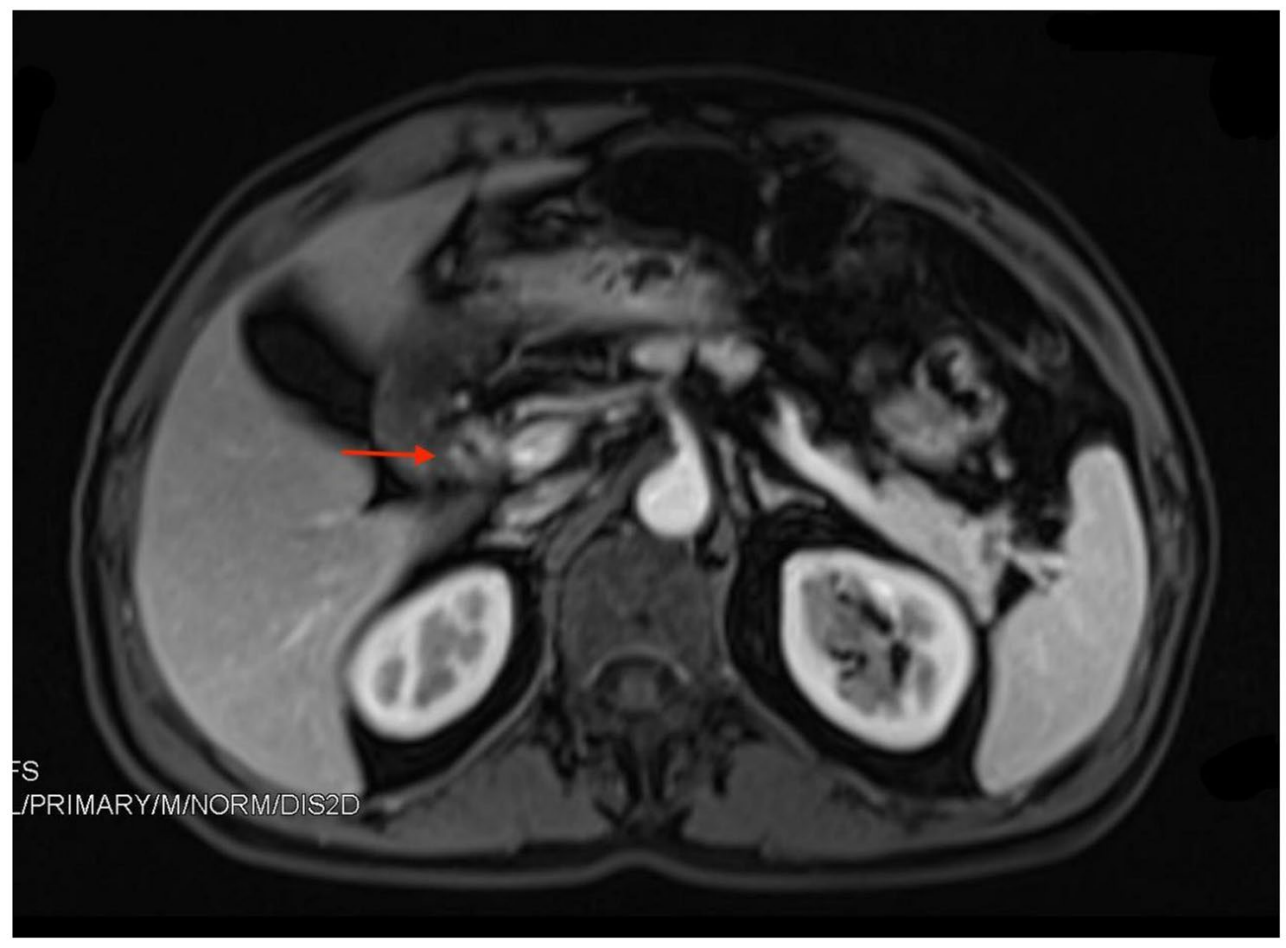
Heterogeneous thicken of the common bile ducts with contrast enhancement on T1-weighted images

Damage to the nerve blood barrier during prior injury could result in increased vascular permeability, which may cause passive diffusion of contrast agents, accounting for the enhancement of TNs [62–64]. In our center, five TN patients with TN underwent cholecystectomy with preoperative MRI images, and none of them had distinct margins. MRI showed heterogeneous thickening of the common bile ducts with contrast enhancement (Fig. 2).

##### Positron emission tomography/computed tomography (PET/CT)

PET/CT has been widely used for the detection and staging of many cancers [65]. It also helps distinguish benign tumors from malignancies. However, this was not a cancer specific examination. Active inflammation often results in false-positive results, and false-negative results have been observed in malignancies with low metabolic activity [66]. Only two cases of TN were reported with PET/CT results, which were reversed. One exhibited no increase in uptake [7], whereas the other exhibited increased uptake [2]. Therefore, the diagnosis of TN cannot be excluded based on positive PET/CT results.

##### Endoscopic examinations

With recent progress in endoscopic technology, endoscopic ultrasonography (EUS), endoscopic retrograde cholangiopancreatoscopy (ERCP), and endoscopic ultrasound-guided fine needle aspiration (EUS-FNA) have been increasingly used in the diagnosis of ampullary and extrahepatic bile duct tumors. Compared to conventional examinations, endoscopic examinations have great advantages in the diagnosis of TN in the bile duct. Several authors have described TN as a homogeneous hypoechoic mass with a clear margin on EUS [57, 58, 67–69]. Intraductal ultrasonography (IDUS) could get much clearer views of the TN located at cystic stump [57, 70].

#### Pathological biopsy

**Fig. 3:**
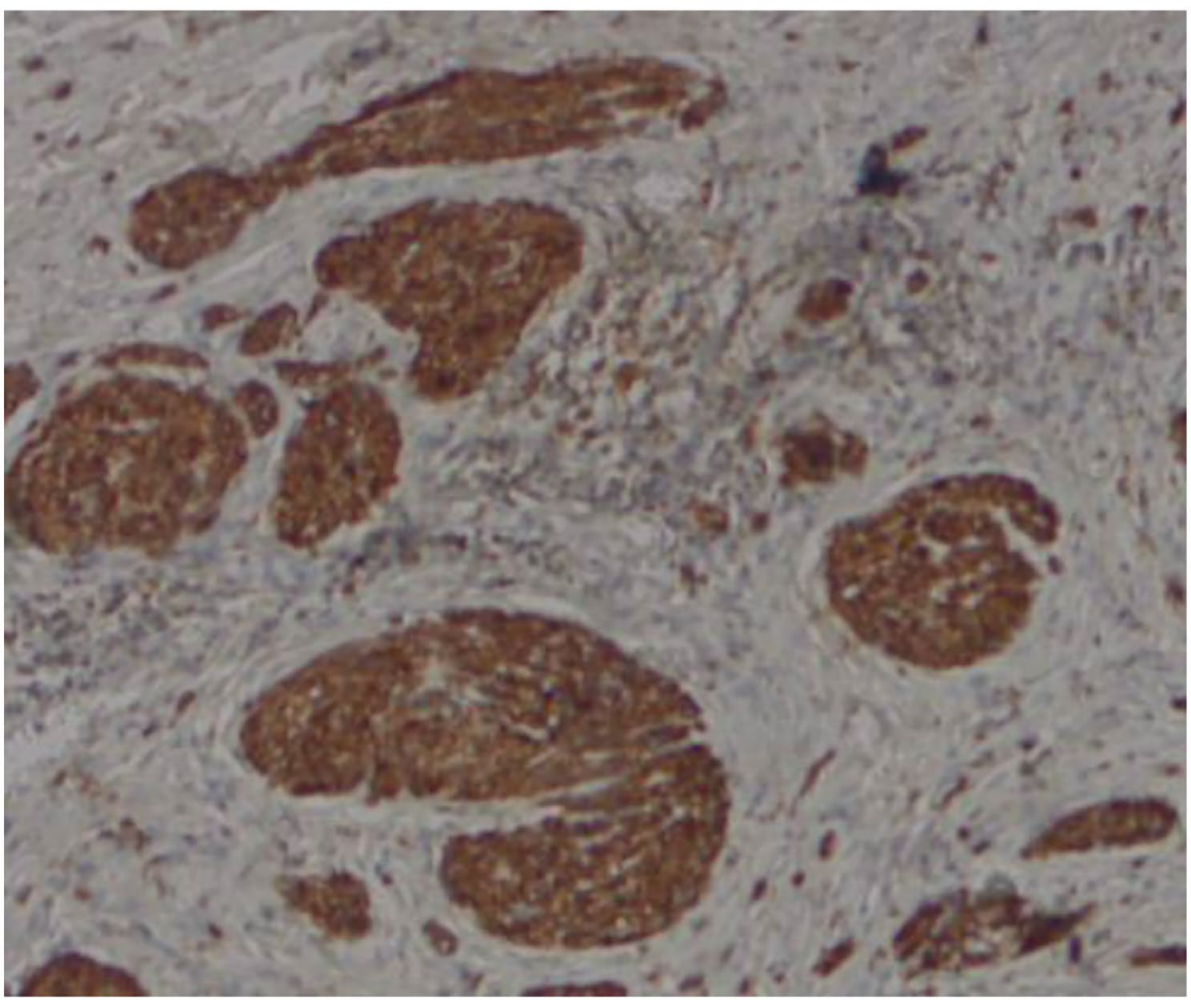
Proliferation of nerve fiber stained by immunohistochemistry of S-100 protein

Some patients with TN can be diagnosed on the basis of their past history, clinical manifestations, and imaging examinations. However, patients with severe symptoms that are difficult to distinguish from malignant tumors require pathological examination to confirm their diagnosis. ERCP enables doctors to observe lesions under direct vision [71, 72]. Yasuda et al. and Toyonaga et al. reported that TNs were covered by normal bile duct mucosa during endoscopic cholangioscopy [58, 67]. Hence, superficial biopsy of the tumor may fail to confirm the diagnosis of TN, and EUS-FNA is useful for obtaining deep tumor tissues, which could improve the accuracy of diagnosis. Microscopically, it is a disorganized proliferation of axons with Schwann cells and fibroblasts in collagenous stoma, which stains positive on immunohistochemistry for S-100 protein (Fig. 3).

## Management

**Fig. 4:**
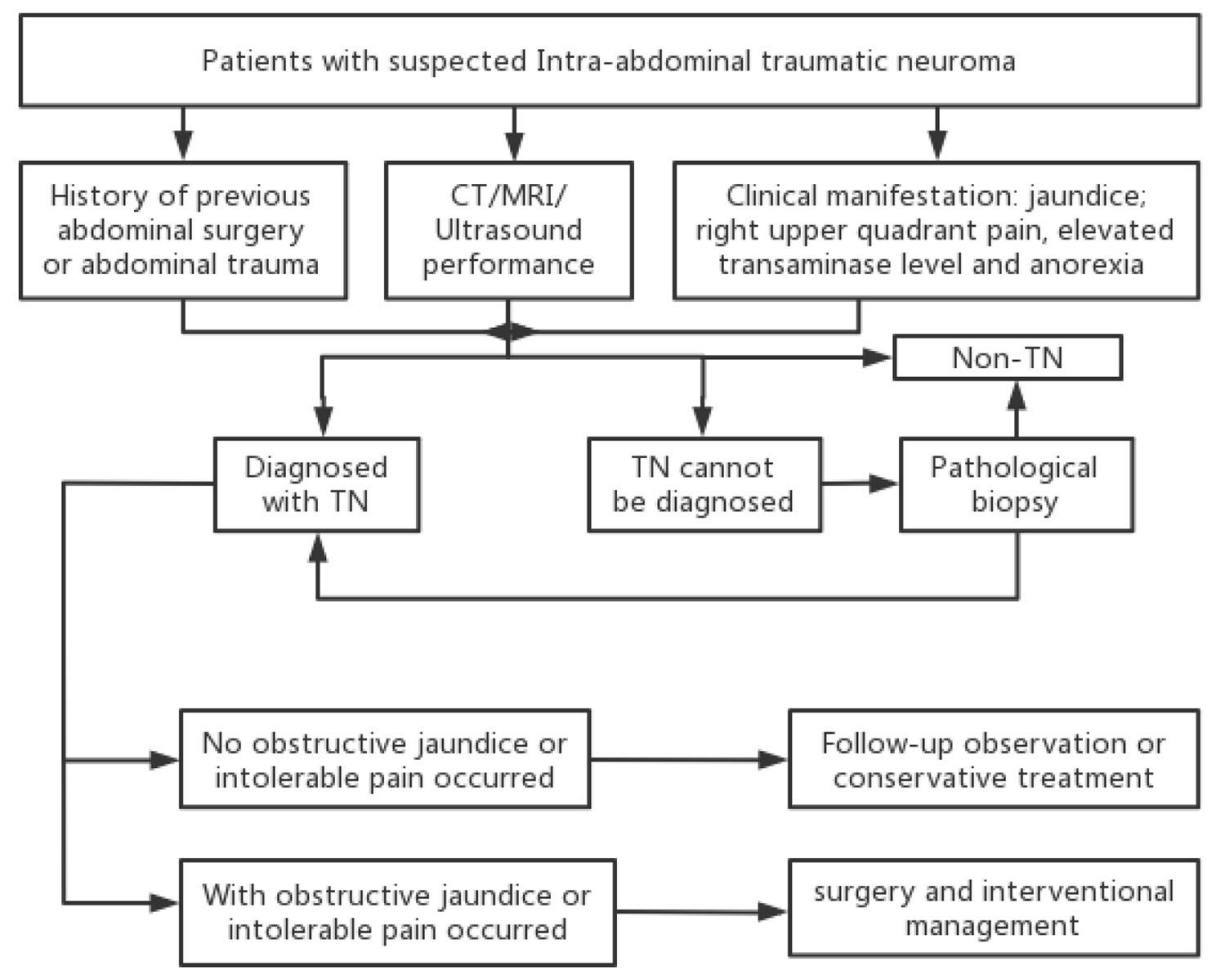
Flow chart of diagnostic and management options for TN.

A flow chart of the diagnostic and management options for TN is shown in Fig. 4. The management of TNs should differ according to their location and symptoms. Regular follow-up is recommended [58, 67]. Regarding patients who only developed abdominal pain without biliary obstruction, Topazian et al. reported that patients experienced temporary relief of pain after injection of bupivacaine and triamcinolone under EUS-guided, but the security and effectiveness still need further confirmation [73]. Surgery is not recommended because pain can recur after resection of TBNs [73–75].

Surgery and interventional management are the most common treatments for patients with biliary obstructions. Several authors suggested that surgery was optimal choice for symptomatic patients of TBN [15, 16, 24]. Among all cases reviewed in this article, resection of the lesion with hepaticojejunostomy was the most common surgical procedure, accounting for 47.1%. Resection followed by duct-to-duct anastomosis occurred in the second place, accounting for approximately 20% patients. Navez et al. considered hepaticojejunostomy to be the best type of biliary reconstruction, based on the fact that the incidence of TBN was higher in patients who underwent duct-to-duct anastomosis during the first liver transplantation, and as a result, it seemed more likely for TBN to recur after duct-to-duct anastomosis [24]. None of the patients who underwent duct-to-duct anastomosis experienced recurrence during the follow-up. The best method for biliary reconstruction requires further study and more precise evidence. Some patients underwent much more aggressive surgeries, including periportal lymphadenectomy, pancreaticoduodenectomy, and hemihepatectomy, owing to difficulties in distinguishing them from biliary malignancies [1, 16, 28, 76]. Frozen section examination during surgery is useful for confirming diagnoses to avoid unnecessary extensive resections [5, 7, 77]. In addition, almost 20% of patients with TBNs after liver transplantation receive retransplantation for reasons of liver failure or rejection [15, 25].

Interventional management consists of two parts. One is preoperative drainage to relieve jaundice, including endoscopic drainage and percutaneous transhepatic drainage under ultrasound or radiologic guidance. The second part aimed to solve the stricture of the bile ducts, including balloon dilatation and stent placement. However, the effects of the interventional treatments did not improve. Multiple cases reported failure of biliary stenting or balloon dilatation for relieving biliary obstruction in the long term [12, 15, 24–26, 28, 78]. Fibrotic nature and poor compressibility may account for these unsuccessful outcomes. In addition, repetitive invasive interventions may accelerate the formation, resulting in an early biliary structure [78]. As for TN located in the gastrointestinal tract, a few authors considered that it was effective to achieve en bloc resection by endoscopic mucosal resection [8, 69].

## Conclusion

Although TN is a benign lesion, it is sometimes difficult to differentiate it from a malignant tumor. TN lacks the typical clinical characteristics. Therefore, it is necessary to make a comprehensive judgment based on the patient’s medical history, clinical manifestations, and imaging findings. If necessary, needle biopsy can be performed to confirm the diagnosis. Conservative treatment is recommended for patients with TN without biliary obstruction. If biliary obstruction occurs, surgical or interventional treatment is necessary.

## Data Availability

The manuscript contains all data from this study.
